# A Scoping Review of Children and Adolescents’ Active Travel in Ireland

**DOI:** 10.3390/ijerph17062016

**Published:** 2020-03-18

**Authors:** João Costa, Manolis Adamakis, Wesley O’Brien, João Martins

**Affiliations:** 1 Sports Studies and Physical Education Programme, School of Education, 2 Lucan Place, Western Road, Cork T12 KX72, Ireland; emmanouil.adamakis@ucc.ie (M.A.); wesley.obrien@ucc.ie (W.O.); 2 Laboratório de Pedagogia, Faculdade de Motricidade Humana e UIDEF, Instituto de Educação, University of Lisbon, 1499-002 Cruz Quebrada—Dafundo, Portugal; jmartins@fmh.ulisboa.pt; 3 Environmental Health Institute, Lisbon Medical School, University of Lisbon, 1649-028 Lisbon, Portugal; 4 Centro Interdisciplinar do Estudo da Performance Humana (CIPER), Faculdade de Motricidade Humana, Universidade de Lisboa, 1499-002 Cruz Quebrada—Dafundo, Portugal

**Keywords:** Republic of Ireland, review, active transport, active commuting, youth, physical activity

## Abstract

There appears to be a lack of existing data that comprehensively summarizes the evidence of children and adolescents’ active travel in the Republic of Ireland. In lieu of this, a scoping review was conducted to map the existing literature (2000–2020) on children and adolescents’ active travel in the Republic of Ireland. A scoping review design extracted a total of 19 publications, which show a consistent focus on the identified population’s active travel patterns, mainly to and from school, mostly self-report and cross-sectional research study designs; however, there are few longitudinal data, intervention and participatory studies. Key issues from these identified scoping review studies are discussed with the potential to better inform policy makers, practitioners and researchers to delineate programmes and strategies for promoting active travel among children and adolescents in the Republic of Ireland.

## 1. Introduction

Regular physical activity (PA) participation reduces the risk of disease, in addition to providing a multitude of benefits that help individuals sleep better, feel better, and perform daily tasks more easily [1]. These benefits can be achieved through a variety of PA modalities, and active travel (i.e., using self-propelled mediums, such as walking or cycling, some or all the way to a destination) can contribute as much as 30% towards meeting the recommended daily levels of PA for health [2,3]. A recent PA review [4] identified positive associations between active travel and health outcomes across 68 studies. Specifically, active travel through cycling was clearly linked with improvements in cardiorespiratory fitness.

In terms of PA participation, children and youth, however, are failing to meet the recommended guidelines for health [5,6], and active travel needs to become more integrated in society as an important additional source of PA participation [7]. Compared with other modalities of PA, active travel has the additional advantage of being convenient and free of monetary costs [8]. Yet, data show a significant decline in active travel over the past 30 years such as the prevalence of active travel for children which was almost 48% in the 1970s but had declined to 13% by 2009 in the United States [9], with similar downward trends observed in Canada [10,11], Switzerland [12], the United Kingdom [13], Australia [14,15], and New Zealand [16].

### 1.1. Summary of Past Reviews on Active Travel of Children and Adolescents

Past reviews sustain that the existing strength of the evidence for active travel is still debatable, warranting continuing research [17,18,19,20]. To promote walking, Carlin et al.’s [17] review concluded that school-based interventions show meaningful potential. A key supporting argument for school-based active travel interventions is that children and adolescents go to school every day, and this environment is a natural and ongoing opportunity to develop active travel behaviours [17]. Other elements that show good promise in promoting active travel are interventions with a systematic design, including intermittent approaches of short bouts of active travel, as well as other settings [17,18,20]. Moreover, past reviews highlight other key factors that contribute to sustain active travel as a behaviour, such as parental involvement, longitudinal trends of youth as they progress through school, peer relationships, urban safety, distance and schedule convenience [17,20].

In terms of how research on active travel is conducted, a large portion of the research is conducted with cross-sectional designs, primarily from walking-based research, and using self-reported data from children and youth [20]. Most of this research also tends to be developed in Europe. Apparently, research with adolescents falls short compared to that with children [17]. Saunders and colleagues [19] argue that there appears to be no standardised way of addressing active travel, where Schoeppe and colleagues [20] state that: “The definition of active school travel varied in terms of frequency and duration of active travel, and journey to and/or from school. Moreover, most included studies did not employ reliable and valid active travel.” (p.317).

### 1.2. Overview of Irish Data on Active Travel of Children and Adolescents

For clarification purposes, the island of Ireland comprises two different national jurisdictions, i.e., Northern Ireland which (along with Scotland, Wales, and England) belongs to the United Kingdom, and the Republic of Ireland as part of the European Union. Each jurisdiction has a respective government, hence its own ministries.

Most recent nationally representative data across the island of Ireland (*N* = 6651; age range 10–18 years old; 53% female) indicates that 42% of primary and 40% of secondary school children self-report walking or cycling to and from school [21]. In comparison to the previously disseminated active travel data [22], this represents an 11% increase in active travel among Irish primary school children since 2010 [21]. In combating the widely accepted age-related decline in PA participation [23,24], specifically during adolescence [25], it is concerning, however, that the most recent 40% figure of secondary school children actively commuting [21] to and from school in Ireland has not increased since 2010 [22]. It could be argued, based on the data of Woods et al. [21] that there is a lack of safe places to crossroads, and the distance to schools are significant barriers preventing Irish adolescents from increasing their levels of active travel.

At a government policy level, in the Republic of Ireland, it has been promising to observe the National Physical Activity Plan [26]. This policy specifically addresses under action area four of the “Environment” that the promotion of active transport is one of the most practical and sustainable ways to increase population levels of PA. For example, one of the specific action four areas from this National Physical Activity Plan is to ‘ensure that the planning, development and design of towns, cities and schools promotes cycling and walking…’ (p.24). While gender and location (urban versus rural) inequalities in active transport are still in existence for Irish children and adolescents [27], the data from Woods et al. [21] has put the promotion of active travel on the map for this population, and recommends that the island of Ireland must now set a realistic and meaningful target for increasing the percentage of children and adolescents walking and cycling to school between 2019 and 2027.

### 1.3. Purpose of This Review

To our knowledge, no review has been published that comprehensively summarises the evidence in relation to children and adolescents’ active travel in the Republic of Ireland. This raised our research question of: “What is the nature and content of research on the active travel of children and adolescents conducted in the Irish context, and what type of implications are (not) being addressed?” Therefore, the purpose of this scoping review is to undertake the following: a) to map and summarize the existing literature findings from the past two decades (2000–2020), specifically relating to contextual study factors, such as age and gender, for active travel in children and adolescents from the Republic of Ireland; b) to document the existing study design, supporting theories, measurement protocol and prevalence of active travel in these identified scoping review studies; and c) to identify current barriers, gaps and areas of opportunity for active travel promotion in the literature. This scoping review can contribute to mapping the key concepts underpinning the active travel research field and evaluating the specific types of evidence-based data available [28]. Ultimately, this scoping review study has the potential to better inform policy makers, practitioners and researchers in order to delineate programmes and strategies for promoting active travel among children and adolescents in the Republic of Ireland.

## 2. Materials and Methods

Given the aforementioned aims, the selection of a scoping review process was identified as the most suitable methodological approach to undertake this study, following relevant literature on the methods of conducting this type of review [28,29]. Guided by the research questions presented above, the following evidence-informed scoping review framework, as proposed by Arksey and O’Malley [28], was implemented:

1. Identifying relevant studies—a PubMed and Scopus database search was conducted. While these different databases provided existing published research evidence, Google Scholar and the Open Access to Irish Research database (RIAN) were also used in the context of this scoping review for identifying outstanding grey literature, such as theses, policies and reports.
2. Study selection—by screening and assessing the data based on the inclusion and exclusion criteria, a set of publications was filtered down to a final selection of 19 studies eligible for the current review.
3. Charting the data—each relevant document was screened and summarised, as the research team achieved consensus on the final list of references for the research.

Each of these three identified stages are described and justified in further detail below.

### 2.1. Establishing Search Terms and Criteria to Identify Relevant Studies

The design of the literature search strategy started with breaking down the research question into an initial set of keywords, such as “active travel”, “Ireland” or “Irish”, “children”, “adolescents” or “adolescence”. With supporting evidence from the literature informed by previous reviews [17,18,19,20], further keywords were added, namely “active transport” or “active commuting”. Having selected the search tools and terms, the chosen databases’ advanced search options and different combinations with Boolean operators (e.g., (“active travel” OR “active transport” OR “active commuting”) AND (“youth” OR “children” OR “adolescence” OR “adolescents”) AND (“Ireland” OR “Irish”)) were employed for the selection of potentially relevant documents.

The main objective and inclusion criteria of this search strategy was to collect all potential sources that specifically investigated and reported elements of active travel as PA of children and adolescents in the Irish context, across a range of publications (e.g., theses and dissertations, statistics, research and policy papers, research reports, conference abstracts and proceedings), regardless of the methodological decisions employed. As the research context for three of the authorship team relates to the Republic of Ireland, and considering that some works presented data in aggregate for the whole island of Ireland, it was decided that only publications with a clear presentation or breakdown of data from the Republic of Ireland were to be included. Finally, an a priori timeframe for the charting of included documents was set to the last two decades, specifically as it appeared that the oldest study was published after 2000. As such, the search strategy was conducted between December 2019 and January 2020 to capture the most recent publications from the last two decades.

As for exclusion criteria, broader concepts such as “independent mobility” were not considered as part of this scoping review, as this research domain typically comprises more elements beyond active travel, such as free play (e.g., [30]). Also, active travel literature from fields outside of the PA domain (e.g., earth sciences such as geography) was excluded.

After establishing essential search terms and criteria, the research team proceeded to extract documents. The choice of the Scopus and PubMed databases sought to include core research fields to the topic of study, namely social sciences and sport/health sciences. With the screening of each document, attention was given to the reference list, specifically in order to identify if a potentially relevant document was missed by the initial search strategy.

### 2.2. Study Selection

**Figure 1:**
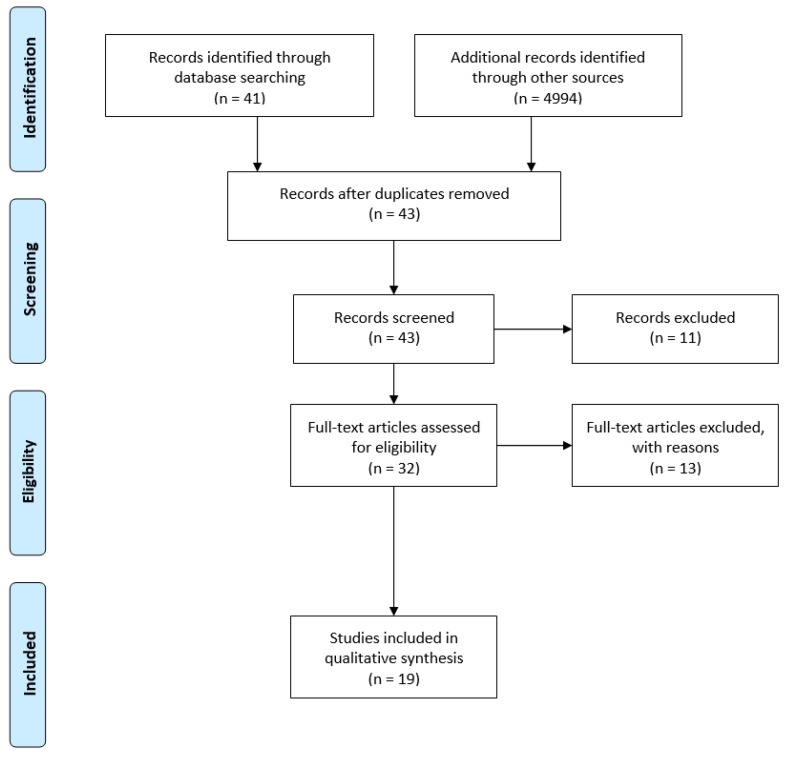
Scoping review flow diagram of reviewed studies from the combined databases and search tools based on the PRISMA protocol. The volume of “additional records identified through other sources” is highly increased from the Google Scholar results which returned 4940 links.

With the input of the identified terms in each search tool and database, the entries provided through Scopus and PubMed had the title and abstract checked. Furthermore, Google Scholar and RIAN were included as search tools to find other potentially relevant grey literature as theses and reports. Based on this process (cf. Figure 1), and after removing duplicates, 43 publications were identified for screening according to the inclusion and exclusion criteria mentioned in the previous stage and reduced to a total of 32 potentially relevant publications for the scoping review exercise. All 32 documents’ data were summarised to assess their full eligibility, leading to a total of 19 studies being included in the review. All the research team were involved in this process. Where questions arose, they were discussed as a team and a collective decision was made. For example, O’Keeffe and O’Beirne [30] present active travel data but because such data is aggregated for all of the island of Ireland, without a specific breakdown for the Republic of Ireland, this document was excluded.

### 2.3. Charting the Data

The process of charting the data was prepared through the design of an online review summary, by assigning each author a balanced set of documents for populating the relevant content. This process started with the 32 potentially eligible documents and concluded with 19 included documents in the review. The evaluation of the document eligibility was facilitated by organising the dimensions and charting guidelines as per Table 1 below:

## 3. Results

An overview of the scoping review process is presented in accordance with the PRISMA protocol as shown in Figure 1.

Table 2 summarises the findings and specifically identifies the study, study design, population/sample, methods used for data collection, the concept of active travel used and theoretical framework, and main findings. The results are presented according to the following themes: methodological characteristics and main findings of the studies.

### 3.1. Methodological Characteristics of the Studies

Overall, very few of the reviewed research items make explicit the theoretical underpinnings that inform the study design, mostly relying on ecological psychology models and frameworks [32,34,43,45]. McMinn et al. [41] present the sole study relying on a social psychology model with the Theory of Planned Behaviour (TPB) [47]. In that study, the Republic of Ireland data were only specifically reported for active travel descriptive statistics, and then aggregated in the Northwest European region to report on the tested TPB constructs. By using an ecological framework, Lambe [43] found similar results and has warned about the importance of differing intention from behaviour. Murtagh, Dempster, and Murphy’s longitudinal study [45] make use of one of Bronfenbrenner’s earlier versions of the bioecological model of human development (1979) [48], considering it to structure the layers of data from the individual (e.g., body mass index, BMI) to the context layers of the immediate settings (microsystems of family, neighbourhood, school, and others). The authors then analyse the associations of those factors with active school travel patterns. Such approach allowed the authors to identify and quantify the: 1) change in distance to school, 2) too much traffic (at baseline), and 3) rural setting (at baseline), as significantly associated to active school travel upkeep, uptake or drop out.

The publications presented in the current scoping review included a minimum sample size of 73 children [39] to a maximum of 16,060 children [36]. Some of the included studies combined data from more than one project [27,40,43,45] subsequently leading to an increased combined number of participants (e.g., Harrington et al. [27,40] with a total sample from both studies ≈30,000 participants). Of special interest is the Census statement document [44], which included commuter data from all Irish primary and secondary students (896,575 commuters). In total, 10 of the identified studies had more than 2000 participants as a sample size.

Most studies focused on children in 5th to 6th class (10–12 years of age) of primary school, while few studies included students at a younger age (i.e., <10 years of age) [33,34,41,45]. Regarding other demographic information provided, more than half of the studies reported participants’ gender (almost even distribution between boys and girls), and a few further studies reported the area of residence and socioeconomic status (i.e., [32,38,39]).

As for the research design, most studies were cross-sectional and used national representative samples [21,22,36,37,44] or non-national representative samples [31,32,33,34,35,38,39,42]. Two studies pertained to the same context and were defined, by the authors, as a community-wide intervention study [43], and a repeated cross-sectional study of a natural experiment [46]. Only one piece of research used a nationally representative sample in a longitudinal study [45].

In terms of data collection procedures, many studies used self-reported questionnaires (only) to assess the active travel data of children and adolescents [31,33,36,37,38,41,43,44,46]. Mode of transportation, frequency, duration and distance were indicators often asked. Other studies used mixed methodological approaches, such as questionnaires and interviews [45]; questionnaires and consultation with young people [39]; and questionnaires, interviews and workshops [34]. A minority of studies used a combination of self-reported data (questionnaires) and objective data (accelerometer and/or pedometers) [21,22,35].

### 3.2. Main Findings of the Studies

The percentage of active travel reported for most studies ranged between 20.0%–40.0%, while some studies reported a higher prevalence of active travel (i.e., 70.0% in Nelson and Woods [32]; 72.4% in Woods and Nelson [42]). In general, there was a higher percentage of children and adolescents who preferred walking, instead of cycling to a destination. The nationally representative studies included found a trend towards the increase of active transport during the last decade, for both children and adolescents [21,22,44]. Two of the intervention studies had no effect on the active travel behaviours to or from school for children and adolescents [43,46].

Based on gender analysis, no active travel differences existed for primary school participants, although secondary school female students were less likely to actively commute than their male counterparts [21,22,31,36,37,40,46], especially when considering cycling [32,36]. It was evident from all studies that active travel is declining with age. For example, Sullivan and Nic Gabhainn [36] reported that the walking percentage was reduced by 3.4% and cycling by 2.4% between the 10- to 11-year age bracket and the 15- to 17-year age bracket. Also, children and adolescents from lower socioeconomic backgrounds were more likely to report active travel to school [36,37].

The main barriers reported in 9 studies that prevented children and adolescents from active travel to various destinations were distance, location (urban vs rural), weather, road, general safety issues, time constraints and heavy school bags. Specifically, for location and distance, it was evident that urban status [21,22,31,37,40,45] and the decrease of distance from the destination [39,45] positively influenced the likelihood of active travel for children and adolescents. The optimal estimated distance for facilitating active travel was 1 mile [31,38].

Finally, only two studies reported on the health benefits of active travel. Murtagh and Murphy [44] found that children who walked or cycled to school had higher daily step counts than those who travelled by passive modes. Clarke and The HBSC Ireland Team [37] concluded that children who reported higher incidences of active travel to school were more likely to report excellent health, as well as increased happiness and overall physical activity levels.

## 4. Discussion

The present scoping review sought to map and summarize the existing literature (2000–2020) regarding active travel amongst children and adolescents from the Republic of Ireland and identify points of future development. This discussion will address the paper’s objectives, namely regarding the a) main findings; b) research methods; and c) current gaps and areas of opportunity.

### 4.1. Main Findings from Research in the Republic of Ireland

Main results showed that the time-trends in active travel, based across the data from various Irish studies, has increased during the last decade; however, in terms of modality, a significant decrease in the proportion of children and adolescents (especially females) cycling to school was noted. The reported levels of active travel in the current scoping review were similar to those of other western countries (e.g., [10,12]), apart from the United States [9].

The age-related decline in active travel was evident in all studies identified. In the Republic of Ireland context, older students tended to walk or cycle less, when compared to their younger peers (adolescents vs. children), and this is a consistent trend globally [6,21,24]. Also, female adolescents had the lowest rates of active travel, which is in accordance with overall PA trends by gender during adolescence [6,49,50]. Apart from the perceived barriers highlighted in various surveys, the decline in girls’ active travel levels may be related to their advanced pubertal maturation compared with boys, though the relative importance of biological and environmental influences on general PA remains unclear [51]. Considering that most studies focused on children above 10 years of age, these studies do not span across both childhood and adolescence, and there is little empirical evidence on the active travel levels of children below 10 years of age. It is possible that the decline in active travel commences in early childhood, even before adolescence, so more longitudinal studies targeting younger children are warranted.

An interesting finding was the particularly low levels of cycling noted in all studies, usually below 3% [21,22,43,44,45,46]. While parental safety concerns and distances travelled are barriers to children’s active travel [21,22,39], other factors include children’s lack of competence in terms of cycling [31,52]. Since active travel through cycling is linked with improvements in cardiorespiratory fitness [4], more high-quality intervention studies, such as longitudinal research study designs with robust methodological procedures could be developed to promote children’s cycling skills, active travel pursuits and the dissemination of evidence surrounding the existing health benefits.

The major barriers that prevented Irish children and adolescents from active travel to various destinations were distance, location (urban vs. rural), and general safety issues. The optimal estimated distance for facilitating active travel was 1 mile [31,38], while most of the adolescents who perceived distance as a barrier to actively commuting lived further than 2.5 miles from school [31]. Similarly, in Switzerland, the large prevalence of children living within 1 mile of school did not change significantly between 1994 and 2005, and this contributed to the relatively high proportion of children actively commuting to school [12]. Also, children and adolescents living in rural areas reported lower active travel levels, mainly because the infrastructure has not been adequately developed, leading to an increased popularity of motorized transportation in those areas [34].

### 4.2. Trends and Contributions of the Active Travel Research Methods in the Irish Context

Of the 19 references included and analysed in this review, a large majority had a cross-sectional research design (e.g., [33,42]), while only a few adopted a longitudinal (e.g., [45]) or intervention approach (e.g., [43]). Most of this is aligned to what previous reviews have observed regarding the research methods on active travel [17,18,19,20]. Given these findings and the need to have quality evidence-based protocols towards the promotion of active travel (walking and cycling) in young people, future research should consider the importance of longitudinal randomized controlled trials [53,54,55]. Cohort studies might be of critical importance to further understand the patterns and predictors of change for active travel throughout childhood and adolescence. Cross-sectional and large-scale representative studies, that use self-reported data collection methods might also be an efficient approach to the continuation of active travel surveillance and identifying the possible associated factors at population level. A distinctive feature found in many of the analysed studies from this scoping review is the inclusion of nationally representative samples (e.g., [21,22,37,45]). Even though, in the last decade, there have been some national studies carried out in the Republic of Ireland (e.g., [21,22]), the vast majority of this research has been with secondary-level adolescents, requiring more of a research emphasis to be placed on primary school children (e.g., [33,39]), and particularly under the age of 10 where data are clearly missing.

As for the data collection procedures, many studies focused on the use of self-report questionnaires (e.g., [31,36,43]). Self-reported data may be subject to social desirability bias [56], and this limitation has been acknowledged in the analysed studies. A few, however, have used a combination of self-report questionnaires and interviews/workshops (e.g., [34,39,45]). Daniels et al. [39] and Lambe [43] align in their need for more qualitative methodologies to better explore the experiences of children and adolescents. Inclusively, Daniels and colleagues [39] demonstrated that participative research methodologies on active travel can be implemented successfully with primary school-level children. A mixed methodology approach for measurement procedures might be more suitable to further understand the perspectives of young people and their parents on active travel, as well as to give young people’s voice and co-construct meaningful solutions to increase their active travel behaviour [57]. A limited number of studies used objective PA data, such as accelerometers or pedometers [21,22,35]. This specific approach might be suitable to capture PA data related to walking, but not cycling. Therefore, future studies should be creative in their technological-based strategies (e.g., Global Positioning System (GPS) use) to better capture objective cycling behaviour.

At a deeper critical level, the reviewed research also refers to issues surrounding research quality. Lambe [43] highlights the critical concerns of: 1) avoiding type 2 errors by stratifying the analysis according to criterion distances and gender for walking and cycling; 2) enhancing data validity to include a “preferred type of active travel” item (given differences between actual and preferred); and c) more accurately measuring active travel when using the Children’s Sport Participation and Physical Activity (CSPPA) [22] self-report survey. Nelson and Woods [32] also consider that research should focus on the perceptions of specific characteristics of the environment related to contextual awareness, such as the ‘presence of pedestrian crossings’, rather than generic statements relating to ‘perceptions of pedestrian safety’. These considerations from Nelson and Woods [32], and from Lambe [43], resonate with Saunders and colleagues [19] on issues relating to active travel definitions and measurement. As discussed above, most of the reviewed studies addressed active travel related to school, based on the recurrent fact that most children and adolescents go to school every day [37]. However, this observation might be too narrow an approach, as school-related active travel mainly contributes to overall quantities of PA participation [2,3], and only a minority of the research analysed in the present review included active travel from a broader perspective beyond the school commute or explicitly considered other modes of active travel used by children such as scooters and skates.

While national policies (e.g., [26,58]) and local authorities are beginning to show alignment and acknowledge the main research recommendations, one of the primary barriers for the near null effect on active travel in the two communities found by Lambe [43] and colleagues [46] was parental gatekeeping. According to the author(s), attenuating the secular trend for the declining levels of active travel to school presents a considerable challenge for local authorities in Ireland. Woods et al. [22] previously stated that active commuters who live in urban areas tend to be more involved in sport and PA, however, overall government departments of transport and sport need to work together to address the issue of non-participation in sport and active commuting. This further concurs with Delaney et al.’s [38] findings, specifically that active participants in sport and recreation events are more likely to use active travel.

### 4.3. Gaps and Areas of Opportunity for Active Travel Research in the Irish Context

The previous discussion sections highlighted essential issues from the reviewed documentation, as the need to use mixed and more robust methods, the overall enhancement of methods quality, the need to widen the sample demographics in the younger cohorts, or considering the issues of definition and measurement of active travel not only to other destinations than school but also considering other mediums of active travel than walking and cycling.

To a much lesser extent, only five [32,34,41,43,45] of the analysed documents in this scoping review highlighted their theoretical underpinnings, raising this as a notable area of opportunity in active travel research. Those works provide examples of contribution to their main theories and can extend the impact towards increasing levels of active travel with children and adolescents. McMinn et al.’s [41] study is an example of how this might be more clearly integrated by using the TPB [47] as a model that explains an intention (represented by the motivation and willingness) towards a given behaviour. According to Ajzen, a behaviour intention is informed by the interconnection of the attitude (referring to the affective evaluation of the relevance of the behaviour), the subjective norm (referring to the perceived positive or negative social pressure on the behaviour) and the perceived behavioural control (referring to the perception of how successful the behaviour can be in a given context). However, McMinn’s study did not present this specific data for the Irish population and, instead, aggregated it in the Northwest region.

While TPB is indeed a valuable theoretical framework to explore the individual attitudes, in the Irish context there is more awareness to ecological frameworks which help to explain the relation between the person and the environment through agency. Two such frameworks are those of Gibson’s Affordance Theory [59], which was not considered by any of the reviewed studies, and Bronfenbrenner’s Bioecological Model of Human Development (also presented as the Process–Person–Context–Time model) [60] which was considered by most of the reviewed research.

Building on Murtagh, Dempster, and Murphy’s [45] use of Bronfenbrenner’s model, it must be noted that this framework has been developed and refined from its earlier versions used by Murtagh and colleagues. According to Bronfenbrenner [60], the proximal processes in the micro- and mesosystem are essential to connect the person to the environment on a developmental perspective, but the research reviewed in our study does not explicitly explore what processes are (in)effective for active travel, and mainly asks about patterns, and contextual facilitators and inhibitors. One such process identified in the reviewed literature can be parental modelling (e.g., [43]) but the specific elements of this (and others) proximal process(es) as being (un)successful need to be better explored and explained. Moreover, if active travel is to be promoted as a desired lifelong behaviour, research needs to more strongly consider not only the immediate settings (microsystems of family, neighbourhood, school, and others) but also their mesosystemic interdependence (e.g., school–family) to provide more sustained, and potentially more effective, implications.

At the same time, as explored by Murtagh, Dempster, and Murphy [45], the personal factors need to be addressed to understand why active travel intentions do not fully, or more regularly, translate into actual active travel behaviour. This dilemma is where the notion of affordances from Gibson [59] may be most important, as a given contextual feature might drive away the active travel behaviour in one person but might make it present in another person. In short, Gibson considered that subject and environment are an inextricable behavioural dyad to present the idea that behaviour emerges from the physical properties of the environment in interaction with the subject’s dispositions and resources. For example, while urban children tend to engage more with active travel, Lambe [43] showed that the contextual changes of the communities did not promote the expected increase in active travel which would be theoretically explained due to the affordance as perception of urban traffic did not change. Lambe [43] also showed that rural area children are more likely to identify the lack of resources as a reason (an affordance) to not engage with active travel. This means that the affordances are very much contextual and situational as a child might be able to cycle in a space where confidence and safety are perceived (e.g., neighbourhood) but not perform that ability/behaviour in a more contextually challenging environment, which could explain at a more theoretical level Nelson and colleagues’ [31] findings of low perceived cycling ability in low active travel patterns. Based on Gibson’s [59] theory, and from a research example provided by Kyttä, Oliver, Ikeda, Ahmadi, Omiya, and Laatikainen’s [61] research with a sample of Finnish and Japanese children, it could be hypothesised that affordances related to active travel are strongly dependent on affordances of independent mobility and this might prove to be an effective focus to the future designs of interventions and active travel research in general.

### 4.4. Strengths and Limitations

A strong point of this review is that it has a clear scope as it attempts to draw a picture of the current research and main findings on active travel in Ireland with children and adolescents. We have tried to point out the factors that are important determinants of active travel according to the existing literature, without making selections or excluding any studies because of their lower quality, or for being published in academic outputs (e.g., journals). Additionally, the search and inclusion process (not only databases but also contextually relevant databases as Irish universities’ repositories), which included developing a search strategy in consultation with a literature review expert and having four reviewers for a proportion of the entire source texts, is a strong point.

One limitation of this review is that its scope may not be broad enough because only studies reporting walking and cycling as means of, mainly, school-related active travel were included. Furthermore, most of the included studies focused on children above 10 years of age, as there is little empirical evidence regarding children below 10 years of age. Additionally, the range of data collection and analysis techniques used in the studies under review makes them hard to compare and makes the mixed results more difficult to interpret. As for the data collection procedures, most studies used self-report questionnaires, which may be subject to social desirability bias. A related issue is that this scoping review did not conduct a quality assessment of reviewed sources. The results should, therefore, be interpreted with some caution. Nevertheless, we believe that the current review provides a thorough survey of the available Irish literature on active travel and the range of research conducted into the subject with valuable insights.

## 5. Conclusions

This paper aimed to map the existing documentation (research, reports, policy) addressing children and adolescents active travel with regards to PA in the context of the Republic of Ireland, between 2000 and 2020, through a scoping review.

Although the levels of active travel among children and adolescents in the Republic of Ireland are higher than in other countries, the existing low levels of cycling to and from a destination, in conjunction with distance and safety barriers, are potentially contributing factors which might explain the difficulty in adopting active travel lifestyle behaviours for children and adolescents. To better approach this, policy needs to keep supporting and building on current evidence, but also ensure cross-sector collaboration and guidelines. Future data collection strategies relating to the surveillance of active travel behaviours for children and adolescents in the Republic of Ireland should consider the suggestions to use and enhance robust methodological instruments (accelerometers, GPS tracking devices, validated PA wearable devices etc.), with scientifically rigorous longitudinal research designs. At an underpinning level, research designs need to carefully consider the issues of active travel definition and measurement, and make more explicit use of, and contribute to, the underpinning theoretical frameworks that support active travel research.

## Figures and Tables

**Table 1:** Charting dimensions and guidelines.

Charting Dimension	Charting Guideline
Study Design	Refer if research was intervention, multiple baseline, case-study, etc.
Population/Sample	Refer sample size, key demographics, and highlight if cohort is primary or secondary education level.
Method	Refer if data were self-reported or objectively measured, with the specific tools if relevant.
Active Travel concept(s) and theoretical frameworks	Identify which concept was used (e.g., Active Travel, Active Transport, Active Commuting) and what theoretical frameworks explicitly informed the study.
Summary of Findings	Summarise only disaggregated data related to active travel in the context of the Republic of Ireland.

**Table 2:** Review summary

Document	Study Design	Population/Sample (n; % of Girls; Mean Age, Age Range; Other)	Method	Active Travel Concept(s) and Theoretical Frameworks	Summary of Findings
Nelson et al. (2008) [31]	Cross-sectional	*n* = 4,013 adolescents; 48.1% girls; 16.1 years, 15–17 years.	Self-report questionnaires.	Active commuting; Mode of transport, barriers, distance. No theoretical framework mentioned.	33% walked or cycled to school; A higher proportion of males than females commuted actively (41.0% vs. 33.8%); Adolescents living in more densely populated areas had greater odds of active commuting than those in the most sparsely populated areas; Most walkers lived within 1.5 miles and cyclists within 2.5 miles of school; A 1-mile increase in distance decreased the odds of active commuting by 71%.
Nelson and Woods (2010) [32]	Cross-sectional (from Take PART study: PA research for teenagers)	*n* = 2159 adolescents; 47.1% girls; 16.0 years, 15–17 years.	Self-report questionnaires.	Active commuting (cycle, walking); Inactive commuting (car, bus or train); Duration, frequency. Mentions the Social-Ecological theory.	Most adolescents chose active modes of travel (61.3% walked, 8.7% cycled); boys were more likely to cycle to school (15.4% vs. 1.2%) and girls were more likely to travel by car (27.0% vs. 18.3%).
Woods et al. (2010) [22]	Cross-sectional (Children’s Sport Participation and Physical Activity (CSPPA) study, Nationally representative Irish cluster sample)	n = 1275 primary school students; 45% female; 11.4 years, 10–13 years; *n* = 4122 post-primary school students; 52% female; 14.5 years, 12–18 years.	Self-report questionnaires; ActiGraph, accelerometers and pedometers.	Active travel; Type of transport, duration, distance. No theoretical framework mentioned.	38% (31% primary, 40% post-primary) of children and youth walked or cycled to school in 2009; journey durations were on average 15 min for active commuters; No gender differences existed for active commuting at primary school; post-primary females were less likely to actively commute than males (38% vs. 43%, *p* < 0.01); 1% of primary pupils and 3% of post-primary pupils cycled to school; Main barriers: Distance (37% primary, 54% post-primary); Time (13% and 19%); Traffic-related danger for primary (13%); and Convenience for post-primary (8%).
Coulter and Woods (2011) [33]	Cross-sectional	*n* = 605 students; 44% female; 8.8 years, 5–15 years. Other: All students from 1 single, large, urban, mixed primary school in Dublin.	Self-report questionnaires.	Active Commuting (as walking or cycling to school on the previous day); Inactive commuting (traveling by bus or car); Estimation of residential distance from School. No theoretical framework mentioned.	39.9% of children actively commuted to school (37.8% walk, 1.1% cycle); 40.7% of children actively commuting from school (39% walk, 1.7% cycle); 56.6% of primary aged children are driven to school; 28.9% live within 1 km of the school but are inactive commuters; Gender did not predict inactive commuting; Compared with younger children (5–6 years), the odds of inactively commuting for every year increase in age decreases by approximately 24%.
Gahan (2011) [34]	Cross-sectional	*n* = 89 adolescents; 6–15 years; 48.3% female (returned questionnaires) *n* = 44 adolescents; 6–15 years; 47.7% girls (participated in the workshop).	Self-report questionnaires; Workshop; Walkability audit.	Active travel; Type of transport, frequency. Mentions social ecological frameworks.	Most commonly used mode of transport by children and young people: 1. Parents’ car (357 times; 2. Walking (205 times); 3. Cycling (80 times); Most commonly use of walking and cycling is going to school, shop, friend’s house; Main barriers: no place to walk (56%); difficulty crossing the road (57%); drivers do not behave well (60%); neighbourhood is not a nice place to live (35%).
Murtagh and Murphy (2011) [35]	Cross-sectional	*n* = 140 children; 39.3% female; 9.9 years, 9–11 years.	Self-report questionnaires; Objective pedometers for step count.	Active travel; Active commute. No theoretical framework mentioned.	62.1% travelled by car, and 36.4% walked to school; Children who walked or cycled to school had higher daily step counts than those who travelled by passive modes (16,118 ± 5757 vs. 13,363 ± 5332 steps).
Sullivan and Nic Gabhainn (2012) [36]	Cross-sectional (From the national research study of Health Behaviour in School-aged Children (HBSC))	*n* = 16,060 students; 49% girls; 10–17 years. From 3rd class in primary school to 5th year in post-primary school. Other: Nationally representative Irish cluster sample.	Self-report questionnaires.	Active travel; Type of transport, duration, frequency (every day). No theoretical framework mentioned.	Walk: boys 23.9%, girls 23.5%; 10–11 years 26.0%, 12–14 years 23.7%, 15–17 years 22.6%; SC1–2 19.9%, SC3–4 23.2%, SC5–6 27.0%; Cycle: boys 3.7%, girls 0.8%; 10–11 years 4.0%, 12–14 years 2.5%, 15–17 years 1.6%; SC1–2 1.8%, SC3–4 2.5%, SC5-6 3.2%.
Clarke and The HBSC Ireland Team (2013) [37]	Cross-sectional (factsheet)	Sample from the HBSC research study; *n* = 12,661 (10–17 years).	Self-report questionnaires.	Travel to school by walking or cycling for the main part of their journey. No theoretical framework mentioned.	26.5% of schoolchildren in Ireland reported actively travelling to school, 28.1% boys, 24.7% girls; Boys, younger children, children from lower social classes, and children living in urban areas were more likely to report actively travelling to school; Children who reported actively travelling to school were more likely to report excellent health, to be very happy, to be more active.
Delaney (2013) [38]	Cross-sectional	*n* = 2877 participants; 53% girls; 12–20 years.	Self-report questionnaires.	Active travel; Distance to school. No theoretical framework mentioned.	24% used active transport as a means of travel to school; Most individuals, who use active transport, live within 1 mile of their school; The percentages of those using active travel dropped the further individuals live from their respective school; Those who were active in sport and recreation activities appeared to be greater users of active travel.
Daniels et al. (2014) [39]	Cross-sectional.	*n* = 73 children; 60.3% female; 11–13 years.	Self-report questionnaires; Workshop.	Active School Travel (walking and cycling). No theoretical framework mentioned.	Non-active travel = 69.9%; 54.5% who reported they actively travelled do so 4-5 days per week; 86.3% reported owning a bicycle; None of the active travellers reported travelling to school with parents; they were more likely to travel to school with friends compared to children who do not travel actively (59.1% vs. 9.8 %); **Main promoters:** 1. Company/Parents and Community; 2. School infrastructure/School; 3. Distance/Parents; 4. Physical Health/Parents, Self, Health Professionals; 5. Equipment/Parents, Self, School, Government; **Main barriers:** 1. Distance/Community and Parents; 2. Weather/Government and Weatherman; 3. Lifestyle/Parents; 4. Road infrastructure and planning/Government, School, Builders; 5. Strangers/Community, Parents, Government, Builders.
Harrington et al. (2014) [40]	Report (including both longitudinal and cross-sectional reports and studies)	HBSC: *n* = 13,611 (11–15 years; 2013–2014 waves—representative sample). Growing Up in Ireland (GUI) Infant and Child Cohorts: *n* ≈ 9,000 children and their caregivers; (Wave 3 of the infant cohort, followed up at age 5 years); *n* ≈ 7400 children; 2011–2012, from Wave 2 of the child cohort, followed up at age 13 years.	Children and parents self-report questionnaires.	Percentage of children reporting active transport to or from school each day. No theoretical framework mentioned.	Active transportation grade D (meaning 21% to 40% meet the defined benchmark); Data from larger studies provided evidence of children/adolescents succeeding with 20% to 29%; Sex gaps evident for other indicators may not be as obvious for active transport; Children from rural areas were less likely to active commute than their urban counterparts.
McMinn et al. (2014) [41]	Cross-sectional (including 5 countries)	*n* = 136; 8.7 years, 69.9 girls.	Self-report questionnaires.	Active commuting; Walkers. Mentions the Theory of Planned Behaviour.	Republic of Ireland 42.0% walkers (i.e., those participants who categorized themselves as being in the action or maintenance stages, according to Theory of Planned Behaviour).
Woods and Nelson (2014) [42]	Cross-sectional	*n* = 199 adolescents; 42.3% girls; 15.9 years, 15–17 years.	Self-report questionnaires; Objective distance (map-measured).	Distance, Time and Mode of active travel (walk, cycle, car, bus). No theoretical framework mentioned.	Mode of transport: walk 72.4%, car 21.1%, bus 6.5%; Distance travelled by active commuters 1.3 km - perceived distance 1.4 km; by inactive 1.4 km, perceived 2.7 km; Active commuters were accurate in their perception of distance travelled; For passive commuters, the average actual distance (1350 m) travelled to school was significantly shorter than their perception of this distance.
Lambe (2015) [43]	Community-wide intervention study collected at 2 time-points (May 2011 and May 2013).	**Study 1:** Primary Education: 5th–6th class students (*n* = 1457) in 21 primary schools (9 in intervention town 1; 5 in intervention town 2; and 7 in the control town). **Study 2:** Secondary Education: 1st, 2nd, the class students in 15 secondary schools (6 schools in intervention town 1; 5 in intervention town 2; and 4 in the control town).	Self-report questionnaires.	Travel mode to school; Actual and Preferred; Awareness of community interventions on active travel. Mentions ecological models.	**Study 1:** At baseline, 25.6% and 3.7% of the total sample walked or cycled to school; boys were more likely to cycle than girls; Greater proportions of students walked or cycled home from school than to school (39.3% vs. 29.3%); Car was the most common mode of travel to or from school in each town (60.8% and 49.1%, respectively); Overall, the intervention had no effect on active travel behaviour. **Study 2:** 17% of the total sample actively commuted to school and distance was a key factor; 64% of the total sample lived more than 3km from their school and of these, only 7% actively commuted to school; Boys were more likely to engage in active travel to school but car travel was still the most common (62%) and preferred (47%) mode of travel for all; Overall, awareness of the community-wide active travel campaign increased by 13% and 20% in intervention towns 1 and 2.
Central Statistics Office (2016) [44]	Cross-sectional (Census 2016, national population survey)	*n* = 896,575 commuters (546,614 primary commuters; 349,961 secondary commuters). Adult respondents.	Self-report questionnaires.	Self-propelled transport (walking - cycling). No theoretical framework mentioned.	**Primary Education:** Active transport decreased from 49.5% in 1986 to 24.8% in 2016. In 2016, 22% of Irish vs. 38% non-Irish walk; 1% of Irish and 2% of non-Irish cycle. **Secondary Education:** Walking decreased from 31.9% in 1986 to 21.2% in 2016; Cycling decreased from 15.3% in 1986 to 2.1% in 2016; Just over a fifth of secondary students walked to school (74,111) up slightly from 73,946 (0.2%) in 2011, but as a percentage of commuters, down almost 2% since 2011; 2016 saw the reversal of this trend with a 10.5% increase since 2011, bringing the numbers of secondary students taking to their bikes to over 7,000.
Harrington et al. (2016) [27]	Report (including both longitudinal and cross-sectional reports and studies)	Sample from: Ireland’s 2016 Report Card Growing Up in Ireland (GUI) Infant and Child Cohorts; HBSC; Children’s Sport Participation and Physical Activity (CSPPA Plus)/	Self-report questionnaires. Interviews.	Active transportation. No theoretical framework mentioned.	Active Transportation - Grade D (The grade for each indicator is based on the percentage of children and youth meeting a defined benchmark, D is 21% to 40%); 23% males, 25% females used of active transport in a local sample of 2877.
Murtagh, Dempster, and Murphy (2016) [45]	Cross-sectional	Sample from “Growing Up in Ireland” study; Wave 1 *n* = 8502 (9 years); Wave 2 *n* = 7479 (13 years).	Interviews. Self-report questionnaires. Anthropometric measures.	Active school travel (uptake and maintenance; dropped out;); Walking and cycling classified as active; Travel mode. Mentions the Bioecological Model.	Within a 4 years period, active travel decreased from 25% to 20%; More likely to uptake or maintain if living in Urban; Less distance affected uptake and maintenance. Walking: Wave1 = 23.8% to Wave2 = 17.8% Cycling: Wave1 = 1.3% to Wave2 = 2.0%; At 9 years of age 75% of children travelled to school using passive travel modes; At 13 years 66% of students maintained passive commuting modes, 14% switched from active to passive commuting, 11% maintained active commuting, and 9% took up active commuting; Overall, at 13 years, 80.2% of the sample travelled to school using passive modes.
Lambe et al. (2017) [46]	Repeat cross-sectional study of a natural experiment	*n* = 1459 5th–6th class students from all the 21 schools in 3 towns (*n* = 1038 students in 2 intervention towns; *n* = 419 students in 1 control town).	Self-report questionnaires.	Actual and preferred mode of travel to and from school; Awareness of the active travel campaign in school and town; Percentage of children that walk or cycle to school. No theoretical framework mentioned.	**Baseline:** Total sample, car use (60.8%), cycle (3.7%), walk (25.6%); Walk or cycle from school (39.3%), to school (29.3%); Bicycle ownership (>85%); Preference for walking and cycling to school was considerably higher than preference for being driven. **Intervention impact:** There was no overall intervention effect detected for active travel to or from school. To school (Town1: pre 33.9%, post 31.2%; Town 2: pre 28.8%, post 33.0%); From school (Town1: pre 41.0%, post 39.5%; Town 2: pre 37.4%, post 38.4%). Some evidence of an effect for males in intervention town 2 (increase of 14% in active travel home from school).
Woods et al. (2018) [21]	Cross-sectional (CSPPA study - Nationally representative Irish cluster sample)	*n* = 1103 Primary school students, 56% female; 11.43 years (*n* = 3594 Post-primary school students; 54% female; 14.11 years; 45% male).	Self-report questionnaires ActiGraph accelerometers and pedometers.	Active travel and active commuting; Type of transport, duration, distance. No theoretical framework mentioned.	42% primary, 40% post-primary school children reported walking or cycling to or from school; 2.2% reported cycling to or from school; At primary school level, more 6th class pupils reported actively commuting than 5th class pupils (47% vs. 36%); At post primary school level, active commuting peaked during 4th year (61%), but was lowest among 6th year pupils (23%); Main barriers: 1. Not enough safe places to cross the road for primary school students (26%), distance being too far for post-primary students (32%); 2. Heavy schoolbags for primary and post-primary school students (22% and 28%, respectively).
